# Sustainable urban systems: Co-design and framing for transformation

**DOI:** 10.1007/s13280-017-0934-6

**Published:** 2017-08-01

**Authors:** Robert Webb, Xuemei Bai, Mark Stafford Smith, Robert Costanza, David Griggs, Magnus Moglia, Michael Neuman, Peter Newman, Peter Newton, Barbara Norman, Chris Ryan, Heinz Schandl, Will Steffen, Nigel Tapper, Giles Thomson

**Affiliations:** 1 Fenner School of Environment and Society, Australian National University, Building 141, Linnaeus Way, Acton, Canberra, ACT 2601 Australia; 2 Beijing Normal University, Beijing, China; 3 CSIRO Land and Water, CSIRO, Clunies Ross Street, Canberra, ACT 2601 Australia; 4 Crawford School of Public Policy, Australian National University, 132 Lennox Crossing, Canberra, ACT 2601 Australia; 5 Monash Sustainable Development Institute, Monash University, 8 Scenic Boulevard, Clayton Campus, Clayton, VIC 3800 Australia; 6 Warwick University, Coventry, UK; 7 CSIRO, Ian Wark Building (B203), Clayton South, VIC 3169 Australia; 8 Faculty of Architecture and the Built Environment, University of Westminster, 35 Marylebone Road, London, NW1 5LS UK; 9 Curtin University Sustainability Policy (CUSP) Institute, Curtin University, GPO Box U1987, Perth, WA 6845 Australia; 10 Centre for Urban Transitions, Swinburne University of Technology, EW Building, Serpells Lane, Hawthorn, Melbourne, VIC 3122 Australia; 11 Canberra Urban and Regional Futures, Faculty of Business, Government & Law, University of Canberra, Building 7D34, Bruce, ACT 2167 Australia; 12 Victorian Eco-Innovation Lab, Faculty of Architecture, Building and Planning, University of Melbourne, Building 133, Parkville, VIC 3010 Australia; 13 Stockholm Resilience Centre, Stockholm University, Stockholm, Sweden; 14 School of Earth Atmosphere & Environment, Cooperative Research Centre for Water Sensitive Cities, Monash University, Clayton, VIC 3800 Australia

**Keywords:** Cities, Complex urban systems, Knowledge co-production, Sustainable urban development, Trade-offs and synergies, Urbanisation

## Abstract

Rapid urbanisation generates risks and opportunities for sustainable development. Urban policy and decision makers are challenged by the complexity of cities as social–ecological–technical systems. Consequently there is an increasing need for collaborative knowledge development that supports a whole-of-system view, and transformational change at multiple scales. Such holistic urban approaches are rare in practice. A co-design process involving researchers, practitioners and other stakeholders, has progressed such an approach in the Australian context, aiming to also contribute to international knowledge development and sharing. This process has generated three outputs: (1) a shared framework to support more systematic knowledge development and use, (2) identification of barriers that create a gap between stated urban goals and actual practice, and (3) identification of strategic focal areas to address this gap. Developing integrated strategies at broader urban scales is seen as the most pressing need. The knowledge framework adopts a systems perspective that incorporates the many urban trade-offs and synergies revealed by a systems view. Broader implications are drawn for policy and decision makers, for researchers and for a shared forward agenda.

## Introduction

The level of global urbanisation continues to increase with 66% of global population living in cities by 2050, so that essentially all future population growth is projected to be in urban areas (UNDESA 2014). The positive and negative impacts of cities on global and local natural environments (Grimm et al. 2008; Elmqvist et al. 2013), social and economic outcomes (Glaeser 2012; Bai et al. 2014), and human health and wellbeing (Vlahov and Galea 2002), will increasingly determine sustainable development outcomes and the prospects for staying within social and planetary boundaries (Raworth 2012; Steffen et al. 2015). Urban contributions to climate change, and the need for transformative mitigation and adaptation, are similarly well documented (Seto et al. 2014; Revi et al. 2014; Watts et al. 2015).

As much urban growth is still to come, there is an opportunity to significantly influence sustainable urbanisation through decision making at local, metropolitan, regional/sub-national and national levels. However, cities are complex, dynamic systems and decision making needs to be supported by relevant knowledge and identification of flexible options and pathways. This calls for an enhanced role of science and scientists in urban policy, planning and management processes (McPhearson et al. 2016a). Researchers can contribute through collaborative knowledge development with urban stakeholders, capturing and translating learning for decision makers in a more systematic way, and facilitating innovation, evolutionary co-design and adaptive management of our cities.

Such collaborative effort has been quite common at local spatial scales and within individual sectors—in the urban context typically at precinct and building levels (e.g. McCormick et al. 2013). Despite some examples (e.g. Balducci et al. 2010; Albrechts 2013), knowledge co-design and co-production approaches are less common at the broader metropolitan scale, and across multiple sectors. Yet decisions at this broader scale have major implications for sustainable development both in their own right, and in setting the context for initiatives at smaller ‘within-city’ scales. Metropolitan decision making also needs to include implications for adjacent regions and more distant impacts (Seto et al. 2012; Seitzinger et al. 2012); and can learn from comparative city and case study analysis across multiple jurisdictions and locations (Seitzinger et al. 2012), taking account of their different urban characteristics (Seto et al. 2010).

This article describes the first stages of a collaborative research, policy and practice co-design process (the second section “The co-design and co-production process”), and summarises the outcomes to date of applying this process (the third and fourth sections “A knowledge framework for sustainable urban development”, “Insights on Australian urban issues from the co-design process”). It initially focusses on the Australian context, but with the intent of contributing to broader international efforts, including the Future Earth Urban Knowledge Action Network (Future Earth 2016). Our overall objective is to better support urban policy and decision making through a more holistic, participatory, systematic and sustained approach to knowledge development and use.

The original contributions of the initiative to date have been to

- extend the scale and scope of the urban co-design process to encompass multi-scale, cross-sector, and multi-agent connectivity and decision making, in support of more integrated, evolutionary and transformational change;
- develop a shared knowledge framework through the co-design process supplemented by insights from the international literature; and
- identify through co-design some high leverage focal areas that are essential for urban sustainability, and related trade-offs and synergies at various scales, drawing initially on the experience of multiple Australian cities.

A key premise is that drawing on the experience across cities within a single nation is a useful first step, as these cities will often have broadly consistent context, history and policy settings. This makes it possible to separate the influence of such national factors from the more specific characteristics of individual cities, and provides a firmer foundation for international comparative city and case study analysis and learning.

Australia has a range of urban challenges (Newton 2008; Kelly and Donegan 2015). It is one of the most urbanised countries in the world with 89% of the population living in urban areas (UNDESA 2014, Table 1). Notwithstanding high ‘liveability’ ratings of the major cities (EIU 2015), current and emerging issues for Australia include the continuing growth in population (e.g. Sydney and Melbourne both projected to double in population to over 8 m people by 2061); ageing and inadequate infrastructure; continuing urban sprawl albeit with some moves towards increased density; work locations distant from home; limited public transport investment; growing traffic congestion; decreasing housing affordability; people and infrastructure vulnerabilities to climate change; and socially disadvantaged communities with growing inequalities.

**Table 1:** Drivers influencing urban decisions, which often become barriers to delivery of stated goals (identified in co-design process with stakeholders, based on experience of Australian cities)

Policy and decision drivers	Examples of issues identified that influence actual decisions
**Overarching drivers**	
Extent of shared vision, goals and leadership at multiple levels	Very variable levels of leadership, and of engagement with stakeholders and communities, across levels of government; short-termism of electoral cycles versus the need for sustained long-term planning; unclear translation of goals to local or project implementations, and to agreed indicators of success
Extent of systemic and enabling policy cohesion	Lack of consistent national government direction and coordinated policies and governance across other levels/sectors; including policies to address many of the more specific drivers below, in order to turn barriers into enablers
**More specific drivers**	
Specific urban context (e.g. geomorphology; history of development; etc.)	Extent of land available for new development influences ‘sprawl’ (e.g. Melbourne has more than Sydney); centrally planned decisions legacy (e.g. very strong in Canberra)
Social drivers	Citizens’ consumption behaviours diverge from stated values (e.g. on sharing and waste); growing urban social issues and disadvantage often hidden from view (e.g. income and wealth inequality; unemployment and entrenched poverty)
Environmental drivers	Lack of appreciation of the value of ecosystem services notwithstanding pollution, waste and natural resource systems depletion/degradation; limited investment in green/blue (living) infrastructure
Economic and financial drivers	Difficulty matching economic development (and jobs) with housing locations; greenfield (vs. infill, and especially ‘greyfield’) development easier economically for governments and developers in the short term; business cases do not reflect externalities and life-cycle costs and benefits; problems mobilising financial capital to include sustainability considerations, including value capture; gaps in practice between ‘as designed’, ‘as built’ and ‘as operated’ performance, suggesting better whole-of-life-cycle approaches needed; sustainability accreditation schemes focus more on buildings than the broader scale
Institutional and organisational drivers	Political cycles and influence; difficulty changing a system that is controlled by a powerful minority (incumbents) who benefit from that system; risk averse planning cultures; lack of consistent and coherent policy and governance across levels/sectors; limited governance transparency and accountability
Technology drivers and new business models	Need to open up access and speed up response to high potential but potentially disruptive technologies (e.g. peer to peer systems and collaborative consumption—Uber etc.; crowd funding; ‘B’ Corporations or Social Enterprises); need to integrate technology with social and institutional change, and new ideas of shareholder value
Spatial and temporal scale complexities	Intrinsic difficulty in evaluation and governance of complex cross-scale issues
Urban planning issues, strategies and practices	Traditional planning (and related professions) focus on urban form and design that is often formulaic using old ‘planning manuals’ and neglecting people and ‘place-making’; planning not well connected to urban ‘processes’ and ‘metabolisms’; political lobbying of powerful private interests distorting ‘public good’ planning; economic development considerations override planning principles
Knowledge, innovation and learning drivers	Limitations on data and credible modelling capabilities, especially across various scales and in support of more integrated and transformational change; need for better evidence base to move from local innovation to scaling up, and speeding up, the transfer and translation of ‘solutions’ into diverse local contexts; need to motivate and activate multiple distributed actors for innovation

Australia also has one of the highest and most unsustainable per capita resource footprints in the world (Wiedmann et al. 2015). Its urban consumption and production patterns significantly impact on regional and global resource extraction (Lenzen and Peters 2010). The transformation necessary to achieve low carbon and resilient conditions is significant (Ryan 2013). Yet institutional arrangements, governance and underpinning knowledge are highly fragmented.

As many of these challenges are common to other countries, the findings aim to be relevant to international efforts. The “Discussion and conclusions” section includes reflexive insights from the co-design process that inform policy and decision making, the supporting research processes, and the potential for extension and broader application of the approach.

## The co-design and co-production process

Co-design and co-production of knowledge are crucial if research is to support those trying to manage and influence sustainability (Lang et al. 2012), especially in policy domains characterised by high stakes, complexity, uncertainty and contestation (Dovers 1995). This involves researchers engaging at the earliest possible stage with decision makers and other stakeholders to ensure that knowledge development will be salient, credible and legitimate (Cash et al. 2003). Participatory approaches to framing research questions, engaging with scientific and non-scientific bodies of knowledge, and tailoring research to the needs of users, have a long tradition in transdisciplinary research (Funtowicz and Ravetz 1994; Lang et al. 2012; Cornell et al. 2013). The approach taken in this initiative builds on this research tradition.

During 2014 discussions commenced in the Australian research community on the contribution that a Future Earth Australia program should make to the emerging international Future Earth agenda (Future Earth 2014). A one-day interdisciplinary workshop identified sustainable urban development as a core theme for Australia, and this was reinforced by a two-day Cities in Future Earth Conference sponsored by the Australian Academy of Sciences (Norman et al. 2014). During 2015 researchers from a range of disciplines commenced a co-design process with urban policy makers and practitioners from around Australia. The initial stakeholder focus was primarily (but not exclusively) government agencies and programs. Overall, thirteen ‘policy/practitioner’ stakeholders were engaged in the process, balanced to ensure representation from national, state, city-region and local council levels, and from a variety of Australian jurisdictions. The national- and state-level representatives were identified by direct approaches to the relevant organisations with urban development responsibility, and the local council representatives with the assistance of national associations for local government. The latter included both inner-city and outer-urban councils on the basis that they may have distinctive perspectives. The researchers involved were the co-authors of this article, who themselves have had extensive experience in collaborative urban projects with stakeholders around Australia and internationally. Researchers and stakeholders funded their own participation. This and the approach to stakeholder identification meant that those who decided to participate were likely to be motivated to contribute and this was borne out by the quality of the subsequent stakeholder contribution.

The co-design process started early in 2015 with a one-day workshop of researchers to share insights from their varied perspectives and agree on the next steps including stakeholder engagement approaches. This led to identification and initial contact with stakeholders; a review of current published metropolitan (i.e. whole-of-city) strategies and plans for the national capital (Canberra) and each state capital (Brisbane, Sydney, Melbourne, Hobart, Adelaide, Perth), with a focus on distilling the urban goals and strategic design principles reflected in those plans; a summary of the relevant coverage of current Australian-based collaborative urban research programs; and a first-pass literature review on urban systems and transformation.

This was followed by a series of semi-structured interviews with individual stakeholders and researchers, the results of which were distilled into key themes. The interviews explored their experience and views on current Australian urban contexts, goals and strategies; the practical barriers to and enablers of more sustainable urban development; and real examples of the issues (e.g. trade-offs and synergies) that can arise when taking a more holistic view of urban systems. The interim findings from all the above were brought together in a whole-day joint stakeholder/researcher workshop in late 2015, which tested the validity of the findings to that point, and built on this to explore the framing options, priority strategic focal areas, associated knowledge needs and next steps for a more systems-oriented and transformational approach to Australian urbanisation. Discussions and conclusions were captured throughout the workshop, and the outcomes subsequently validated with all participants. The outcomes also helped focus a second stage international literature review during 2016, which related the Australian findings more overtly to current urban development and related research internationally. The progressive iteration and testing of findings with participants throughout the above activities proved an effective way of developing agreed summary outcomes.

Links back to Future Earth processes were made through presentations on the initiative and initial findings to an Asia-Pacific Future Earth urbanisation symposium in China in late 2015 and a Future Earth Australia workshop in Canberra in 2016.

**Fig. 1:**
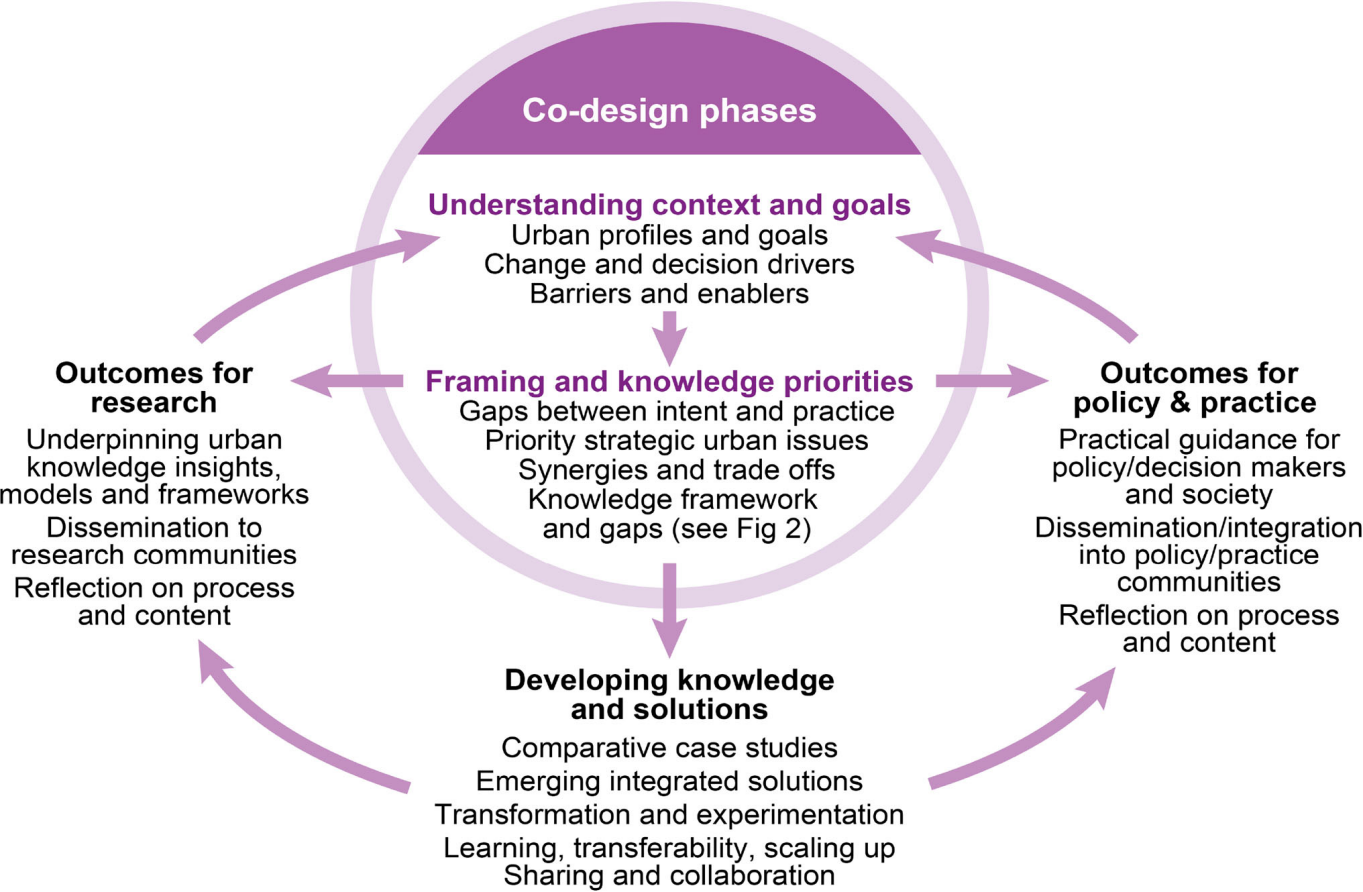
Overall knowledge co-production process for sustainable urban development: developed through, and used in, the co-design process with stakeholders. Two outcomes from such a process are envisaged: (1) practical guidance on policy and practice to assist government agency, utility, private sector, investor and community decision makers at various levels; and (2) insights, frameworks and models that contribute to future collaborative research. The whole process is reflexive and iterative, which is essential when dealing with complex systems

As an overarching approach to guide the above activities we progressively developed and followed the knowledge co-production process at Fig. 1, including some early iterations between phases. The process has three collaborative phases (‘understanding context and goals’, ‘framing and knowledge priorities’ and ‘developing knowledge and solutions’), with outcomes for both practice and research also providing an opportunity for shared reflection and iterative adjustment. The first two phases can be thought of as the co-design element of the overall co-production process, and have been the primary focus of the collaborative work reported in this article.

While developed jointly with stakeholders as part of the co-design process, it is also compatible with other transdisciplinary research and co-production approaches proposed for complex and contested issues. For example the overall phases and their sequence are very consistent with those identified in Lang et al. (2012), Mauser et al. (2013) in the context of Future Earth’s transdisciplinary ambitions, and Grove et al. (2015), Polk (2015) and Frantzeskaki and Kabisch (2016) in the context of sustainable urban development, though each of these uses slightly different terminology to label each phase. There is also a growing literature on specific aspects of urban knowledge co-production (e.g. Munoz-Erickson 2014 on identifying relevant roles of and connections between urban actors; Nevens et al. 2013 on use of urban transition labs to explore innovative approaches; Gorissen et al. 2016 on approaches to accelerating and scaling up transitions). Many of the above sources also helpfully identify detailed steps to work systematically through the entire co-production process, and a range of co-production challenges and good practices, especially for continuing success over a long period of time. Being at a relatively early co-design and framing stage, we have not yet had to face all those challenges, but our approaches have nevertheless been consistent with their recommendations for early stages e.g. an open and inclusive process to facilitate framing, sharing of all information, opportunity for reflexive and iterative thinking, encouraging a diversity of knowledge types and experience, and approaches that facilitate knowledge integration.

In addition Fig. 1 includes under each phase the topics that were agreed as likely to be most relevant for our initiative and the urban challenges it aims to address (e.g. the importance of identifying barriers and enablers to meeting urban goals; and synergies and trade-offs faced by urban decision makers). It was the use of this process, guided by the collaboratively identified topics that led to the outcomes reported in the remainder of this article. The outcomes in the “Insights on Australian urban issues from the co-design process” section were derived directly from the collaborative work with stakeholders; those of “A knowledge framework for sustainable urban development” and the “Discussion and conclusions” sections partially so, but supplemented by insights from the international literature.

## A knowledge framework for sustainable urban development

**Fig. 2:**
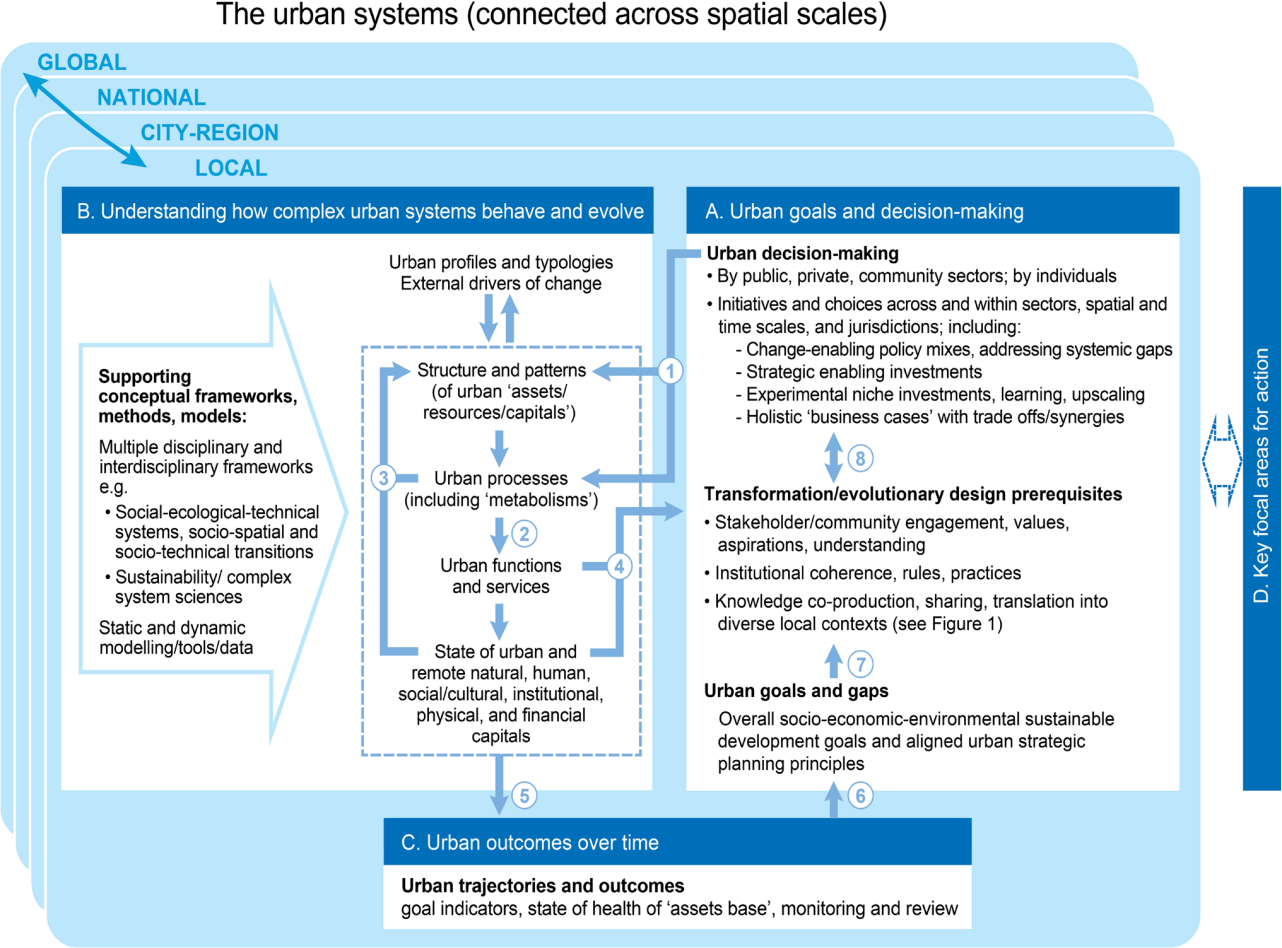
Knowledge framework for sustainable urban development: developed through the co-design process, supplemented with insights from the international literature (Component D is elaborated on at Fig. 3). Note that more than one word is sometimes used to convey a similar meaning, to encompass alternative descriptors from multiple disciplines. Major linkages between components are: (1) urban decisions and choices at many levels directly influence the structure and spatial patterns of urban assets (resources, capitals) at a point in time, and the processes associated with those assets (‘assets’ are here broadly defined to cover human/social/institutional, natural/environmental and built/technical components of the overall urban system; (2) these in turn determine the level and nature of urban functions and services, and, through these, the enhancement or degradation of urban and remote assets over time; (3) autonomous and complex feedbacks take place between these components, often with unintended consequences; (4)/(5)/(6) the actual functions/services experienced and the observed impacts on assets over time, influence future goals and decision making through both informal (and sometimes subconscious) feedback processes (4), and more overt and formal policy review processes (5)/(6); (7) formal goals (such as the UN SDGs and their translation to specific urban contexts) have the potential to drive intentional evolutionary and transformative change; (8) however, to achieve this, urban decision making at all levels needs to consciously engage with and progressively reshape the three fundamental prerequisites for such change; and recognise that flexibility is needed to explore, accommodate and respond to the emergent nature of complex urban systems. These processes are operating at multiple and interconnected spatial and temporal scales, which in practice are further defined by the key focal areas for action that are under investigation

One of the key topics identified in the co-design phases in Fig. 1 is the development of an overall knowledge framework. We have developed such a framework for sustainable urban development (Fig. 2). In this context we use the term ‘sustainable urban development’ to cover not only sustainable resource use and impacts, but also the need for socially just, equitable, inclusive, liveable and resilient development. This is aligned with the scope of the recently adopted UN Sustainable Development Goals (SDGs) (UN 2015).

The framework was developed in part to assist in positioning the findings from this initial co-design process, but we also had in mind the potential for broader and longer-term use. The importance of such a framework is that it can facilitate shared and sustained understanding across multiple disciplines and stakeholders, and assist in more systematically mapping, integrating and translating new and existing knowledge into policy and practice.

While the framework should continue to evolve in use, we have gone to some lengths to make it as robust and well-grounded as possible. The overall structure, and key features of the framework, emerged from the co-design process. This includes the incorporation of multi-scale connectivity, and the need to emphasise the systemic influences *on* decision making by many agents operating at multiple levels, as well as the systemic impacts *of* decisions. This reflects a deliberate focus on developing knowledge that will assist such multi-scale decision making. The framework was also significantly informed by a number of overarching ‘social-ecological’ frameworks and systems approaches from the literature (e.g. Pickett et al. 2011; Grimm et al. 2013; Grove et al. 2013; Wu 2014; Díaz et al. 2015; McPhearson et al. 2016b; Bai et al. 2016).

It views sustainable urban development through several interdependent components which also operate and interconnect at multiple (local, metropolitan, regional, national, global) scales (Grimm et al. 2008; Pickett and Zhou 2015). The central view (Component A) is that of urban goal setting, decision making and other choices by agents operating at multiple levels, and the associated prerequisites for guided evolutionary design and transformational change. Both formal decisions and informal choices are then transmitted through the complex urban systems and processes (Component B), becoming key drivers of urban outcomes and trajectories over time (Component C). Actual outcomes experienced in turn progressively influence urban decision making and choices, and the associated goals and strategies (Component A).

There are many autonomous or directed push, pull and feedback factors operating within this overall system, and some of the higher levels of these are described in the caption to Fig. 2. It should be stressed that all of the Components (A, B, C) are part of the overall system, so that decision makers are seen as being within, and not merely operating on, the urban system. The key focal areas for urban action (Component D) are the decision areas identified where policy and decision makers can most influence sustainable urban development, and may well vary depending on the local context and scope of investigation. In our case they will represent the key decision areas identified in the co-design process for sustainable development of Australian cities. These could well have relevance elsewhere.

### Component A: Urban goals and decision making

To facilitate transformational and integrated (whole-of-system) strategies, decision making should be guided by urban goals, preferably compatible with (and even translated from) the UN SDGs. The latter include an Urban Goal (Goal 11), but in fact many of the 17 goals and the associated targets, synergies and trade-offs (Nilsson et al. 2016) are relevant for urban decision makers (UCLG 2016). For example, liveability may often be attained at the expense of sustainability, as is the case in Australia (Newton 2012). The translation of such goals and their interdependencies to individual cities and communities, combined with measures of sustainability (Neuman and Churchill 2015) and gap analysis, provide an overall context for assessing urban priorities, and addressing trade-offs and synergies.

The framework encompasses decision making and actions by all relevant agents (public, private and community sector-based as well as individual citizens). In pursuit of innovation and transformation, initiatives can include individual initiatives ranging from smaller-scale experimentation with the potential for subsequent scaling up (Bai et al. 2010), to larger-scale strategic enabling investments (Newton 2007) and systemic change-enabling policies. Holistic framing of such initiatives is crucial to capture key interdependencies, trade-offs and synergies.

The more transformational changes are underpinned by evolutionary design approaches (Costanza 2014) which embrace experimentation, as well as the need for underlying structural changes. This includes progressive alignment of three interdependent change prerequisites: stakeholder ‘values’ (also often referred to as ‘worldviews’ or ‘cultures’); institutional ‘rules’ and ‘practices’; and knowledge (including technologies) translated to local context (Grimm et al. 2000; Beddoe et al. 2009; Gorddard et al. 2016). Urban stakeholders and decision makers are diverse with individual, institutional and political biases contributing to potential conflict and a ‘cognitive dissonance’ barrier to sustainable development (Rees 2010). Stakeholder engagement is therefore required on future aspirations and scenarios (Costanza 2014; Ryan et al. 2015) as well as near-term actions (Ryan 2013).

Also explicitly included is the growing body of knowledge (such as the sources cited in “The co-design and co-production process” section) on how solutions can be co-created collaboratively by stakeholders and researchers, as well as how to make effective use of the experience of others.

### Component B: Understanding how complex urban systems behave and evolve

The framework also shows that decision making needs to be supported by an understanding of how urban systems behave and evolve. This includes an appreciation of the extent to which specific urban profiles (e.g. stage, scale and rate of urbanisation, and urban location, form, function and processes (Seto et al. 2010)), and external human and natural drivers influence urban systems; and whether this suggests that certain urban typologies can be of value in increasing understanding. The city is an open system with many interactions with the region and beyond, so some drivers will be exogenous to the city (e.g. national policies, climate change, migration). The drivers may manifest as either shorter-term ‘pulses’ or longer-term ‘presses’ on the system (Collins et al. 2011).

The specific urban profile, along with decisions and other drivers, shape the highly heterogeneous social, biophysical and physical patterns (spatial and temporal), and processes, associated with the full range of urban assets or resources. Assets here are broadly defined, and can (as noted in Fig. 2) also be described in terms of six ‘capitals’: the five capitals identified by Ellis (2000) (physical, financial/economic, natural, human and social capitals) plus institutional capital. The latter reflects the formal and informal ‘rules’ and governance capabilities that underpin the urban policy and decision-making processes (Platje 2011).

The urban processes can also be social, biophysical or physical. The flows of water, material, energy and nutrient resources in and out of cities are structured under the concepts of ‘urban metabolism’ (Kennedy et al. 2007). These concepts can also be linked to the views of the city as an ecosystem (Bai 2016), which is consistent with suggestions that metabolism approaches could be extended to the impacts on, and role of, ecosystems and social resources and actors (Newman 1999; Newton and Bai 2008; Pincetl et al. 2012).

As ‘systems of provision’ (Ryan 2002, Ryan et al. 2015) the urban processes in turn provide functions and services, and lead to the enhancement, maintenance or degradation of the urban and remote assets over time. These processes and their impacts also feed back into the drivers of change, and the urban structures and patterns, sometimes leading to unintended consequences. These feedbacks may be biophysical (e.g. impacts on local and regional climate) or behavioural (citizen choices and preferences e.g. Schelling 1969 on segregation).

The societal experience of actual services delivered and the progressive impacts on assets may lead through formal monitoring to the review of goals and strategies (Component C), but in practice also exercises a more direct, less formal and evolutionary influence on stakeholders’ expectations, decisions and choices (Component A). However, the investment in long-lived assets can also create physical, social and institutional path dependency and unhelpful ‘lock in’ to current directions (Geels and Schot 2010).

Understanding of these urban systems can be facilitated by a range of useful frameworks and methodologies including resilience and social–ecological systems thinking (Folke 2006; Ostrom and Cox 2010); the view of the city as a combination of complex social–ecological–technical systems (SETS) (Ramaswami et al. 2012; McPhearson et al. 2016b); socio-technical transition theories and management (Grin et al. 2010; Loorbach 2010); and socio-spatial thinking at various scales (e.g. Albrechts (2013) on strategic spatial planning typically at or within the city-region scale, and Brenner and Schmid (2015) on wider socio-economic drivers and ramifications of ‘extended’ urbanisation up to the global scale). It can also draw on complementary disciplines such as urban ecology ‘in’, ‘of’ and increasingly ‘for’ cities (Grimm et al. 2016; McPhearson et al. 2016b; Pickett et al. 2016); and on insights from sustainability science (Kates 2011), complex systems science (Batty 2008) and a range of analytic tools including static and dynamic models at various scales.

### Component C: Urban outcomes over time

The complex system interdependencies can generate many possible urban transition pathways. Alternative urban development trajectories can have very different sustainability outcomes (Bai 2003; Newton and Bai 2008; Pickett et al. 2013), and it is also possible that similar sustainability outcomes can be achieved with quite divergent social, cultural and political characteristics (Ryan et al. 2015). Thus, guiding the realised trajectory becomes critical to achieving goals, while recognising that such complex systems are emergent and not simply amenable to top–down command-and-control approaches. Hence flexible strategies and adaptive management need to be supported by multi-level governance, indicators, monitoring and evaluation processes.

### Component D: Key focal areas for action

Finally, the framework reflects that a set of key focal areas need to be identified, where policy and decision makers have the best chance of guiding sustainable urban transformations. Conceptually these are similar to the city transformation ‘action fields’ identified in WGBU (2016). However, in practice these will always depend to some extent on the context. In our case we are looking at informing a national change agenda across and within major cities in Australia, and the focal areas identified in this context are discussed in the following section.

The framework at Fig. 2 should continue to evolve iteratively through application. As it stands some of the more academic concepts are not familiar to practitioners, and will require translation into language they can more readily relate to, similar to the dual-language approach used by Diaz et al. (2016) for the IPBES framework. Nevertheless it can be used to help position some of the other outcomes from our co-design process, as described below.

## Insights on Australian urban issues from the co-design process

In a first-pass analysis of Australian urban sustainable development the co-design process has identified, for the capital cities, the stated urban goals and related urban design principles, the current drivers of the gap between these and actual implementation, and a number of focal areas with high potential to address the gaps.

### Understanding the gaps between stated intent and actual implementation

As reflected in Fig. 2, sustainable urban development can be guided through agreed overarching goals, potentially translated from the UN SDGs. The review of current Australian capital city metropolitan plans revealed that, although they mostly preceded the adoption of the UN SDGs, they already incorporate a similar broad set of goals and consistent urban design principles to meet these goals (see Box 1 for a synthesis of the principles drawn from the city plans).

**Box 1:** Consistent sustainable urban development planning and design principles (synthesised from current Australian metropolitan strategies/plans and validated through co-design process with stakeholders) indicating a growing consensus

More compact form rather than continuing urban sprawl	
Productive agricultural land and connected landscapes protected	
Polycentric city with distributed activity and job growth centres	
Reduced car dependency, increased public transport, ‘30 min city’	
Place-based mixed-use development allied with transport corridors and hubs	
Mixed-use and more self-contained communities	
More distributed infrastructure (e.g. water, energy, food)	
More self-sufficiency in food, water, energy through, for example, urban agriculture, water sensitive urban design, rooftop solar/renewables	
Water sensitive urban design (WSUD)	
Increased focus on blue and green (living) infrastructure	
Physical and social infrastructure that facilitates diverse social interaction, supporting creative innovation	
Neighbourhoods and entire metropolitan areas that are walkable and cyclable	
Greater housing choice, more compact and affordable housing, more quality shared spaces (public and utility spaces)	
Circular economy with reduced resources usage/waste/emissions and ecological footprint	
Low carbon, climate resilient strategies with emphasis on coherent strategies so that decarbonisation and resilience achieved concurrently	

This is not surprising as the plans reflect urban planning theories and movements that have evolved internationally over more than twenty years. These include the Healthy Cities movement (WHO 2016) and New Urbanism (Congress for New Urbanism 2016) since the 1980s; the Compact City since the 1990s (OECD 2012); and Sustainable Urbanism (Farr 2007), the Ecological/Carbon–Neutral City and Regenerative Cities (Girardet and World Future Council 2010). The more recent of these draw on New Urbanism and Compact City ideas, but emphasise integration with nature, reduced materials usage, waste and emissions, and a restorative relationship between cities and the local and distant natural resources they depend on. In addition the Resilient Cities movement emphasises resilience to major change including (but not only) climate change (Rockefeller Foundation 2016).

Individually these approaches emphasise different aspects of sustainable development, and over time reflect a gradual extension from liveability issues to include sustainability and resilience concerns. Collectively they are aligned to the intent of the SDGs, and the urban design principles summarised in Box 1.

However the co-design process concluded that, while these principles and the underlying goals are reflected in major Australian city plans, there are significant problems in translating them into practice. It identified a number of external and local drivers that currently influence strategy, decisions and action in Australian cities; and how these often become barriers to effective implementation of the goals and principles. These are summarised in Table 1, noting especially the two overarching needs for shared visioning and goal setting, and more coherent and systemic policy setting.

In particular many of the drivers are interconnected; most of the drivers, while they may be influenced locally, are beyond the control of any one jurisdiction; and current institutional and policy settings provide incentives to decision-makers that are often counter to the stated goals. Understanding these drivers is a first step towards developing policy and practice, from national through to local levels, which better support sustainable urban development.

### Strategic focal areas for integrated and transformational change

**Fig. 3:**
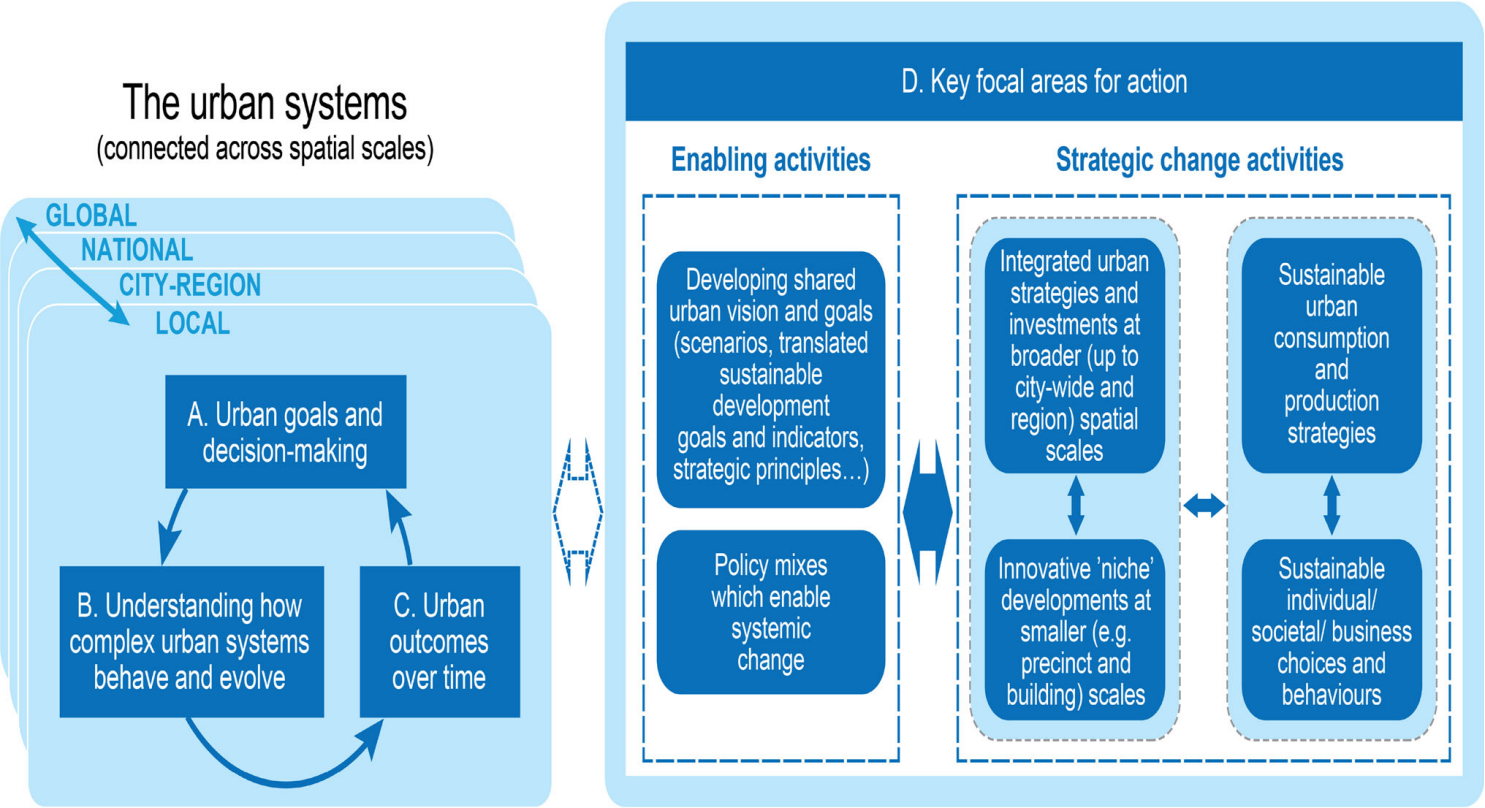
Knowledge framework for sustainable urban development (see Fig. 2), in this case elaborating on the key focal areas for action (component D) identified through the co-design process with stakeholders

The co-design process also identified six strategic decision-making areas that could contribute to more sustainable urban development in Australia (the ‘key focal areas for action’ referred to in Fig. 2, Component D). These are summarised at a high level in Fig. 3.

The analysis in Table 1 identified a need to develop shared urban scenarios, vision and goals at national, city-region and local levels, and more systemic change-enabling policies, in order to address the range of identified barriers. These two focal areas were therefore seen as overarching enablers for integrated and transformational change.

At the next level down the focal areas identified ranged from larger metropolitan-scale strategies and investments, to precinct and building design decisions, and influencing more sustainable business and citizen choices.

Initial consultations in the co-design process tended to emphasise the more experimental and locally driven renewal and reinvention initiatives, typically at neighbourhood, precinct and building levels, that can fully take into account local context and community needs and aspirations, and also demonstrate the potential of new approaches for future scaling up and transfer.

It was also recognised that the value of such initiatives can be enhanced by complementary strategies encouraging more sustainable consumption and production choices and behaviours by individuals and communities (Ryan 2013; Newton and Meyer 2015) and businesses (e.g. Ellen Macarthur Foundation 2013 on the circular economy and industrial symbiosis).

However, the ensuing discussions increasingly focused on the identification and framing of strategic issues and investments with additional potential to drive holistic and transformative change. These were often at the broader spatial scales, from the precinct upwards to the whole-of-city and metropolitan region, and across sectors. Developing holistic solutions at these broader scales was seen as doubly important as: (1) they can make important contributions to sustainable development in their own right; (2) planning and investments at these scales provide a clearer direction within which precinct and building scale urban development, and sustainable consumption and production initiatives, equally essential to overall transformation, can proceed with greater confidence.

As a future knowledge development priority, the focus on such issues also reflects that (within Australia at least) there is already considerable investment in collaborative research and modelling at the sectoral and/or building and precinct scale (e.g. current programs on Low Carbon Living, Water Sensitive Cities, Clean Air and Urban Landscapes; Housing and Urban Research; Sustainable Built Environment). However, while many of these take systems approaches, none have a whole-of-urban-system charter.

Specific strategic focal areas identified across scales are summarised below.

*Whole*-*of*-*city, city*-*region and related cross*-*sectoral strategies*: These encompass:

- clarifying the roles of the city in the development of the broader region (Neuman and Hull 2009), including concepts of the ‘polycentric region’, and recognition that urban and rural systems are strongly coupled;
- clarifying the relative value of development and infrastructure investment in the inner *vs* middle *vs* outer suburbs, including approaches to the ‘polycentric city’, central vs distributed industry and job locations, and the desire to reduce urban sprawl; and
- resolving sector and cross-sector issues that traverse the city scale, including new approaches and strategic investments in key interconnected metropolitan infrastructure and service networks: energy, water, transport and food (e.g. Newton 2012, 2013). This includes issues such as centralised *vs* distributed energy, water and food infrastructure; understanding cross-sector interactions, trade-offs and synergies (e.g. the urban food–energy–water nexus (GIZ and ICLEI 2014), and the urban planning/infrastructure–transport–health nexus (Bai et al. 2012)); and reinforcing links from these to local and city-wide decarbonisation and climate adaptation strategies.

*Outer-urban and peri*-*urban choices*: Here, land-use and infrastructure decisions need to reconcile trade-offs between the differing interests of communities, governments (state and local), developers, local industry (including agriculture and horticulture), and water catchment and natural environment managers. Decisions should be guided by alignment with whole-of-city and city-region strategies and by more holistic economic and valuation models (e.g. recognising the full cost of urban sprawl in greenfield developments (Trubka et al. 2010)). The fact that urban sprawl persists, despite explicit city goals to the contrary (an example of the playing out of the barriers identified in Table 1), means changing policies, incentives or transfers between the city as a whole and its outlying suburbs.

*Urban renewal and intensification decisions*: These range from renewal of urban corridors, CBDs, suburbs and suburban centres down to individual precincts. They can integrate with and facilitate the broader ‘whole-of-city’ directions. Core strategies are increasing density (Newman 2014), and reducing automobile dependence in ways that recognise the distinctive urban planning needs of a city’s three different ‘urban fabrics’ (Newman and Kenworthy 2015: Chapter 4) i.e. the physical elements, and social and business functions that best match the walking-city (typically up to 2 km around centres), the transit-city (based primarily on tram, train and bus corridors), and the automobile-city (between and beyond the other two fabrics). Compared with the redevelopment of ‘brownfields’, established middle suburbs (greyfields) present a particular intensification challenge (Newton et al. 2012; Newton 2013). It was also stressed that ‘people and place’-centred design, including social and living (green/blue) infrastructure, is crucial. Standard urban design typologies, including approaches to intensification, need to be translated to fit diverse local contexts reflecting climate, topography, environment and socio-cultural needs. Moreover, spatial practices such as urban planning and urban design should take into account that sustainability is about urban processes as well as urban form (Neuman 2005). The above means addressing community aspirations; incorporating physical, social, and culturally attuned architectures that encourage sustainable behaviours; and anticipating risks associated with intensification (e.g. irrigated green infrastructure to address urban heat island effects (Tapper et al. 2014)).

*Temporal scale challenges*: In each of the above spatially differentiated domains, conceptual and practical issues were identified in handling timing and sequencing of decisions. Examples included how infrastructure investment should be staged, and the extent to which it should lead or lag residential development; the need for whole-of-life-cycle costing of investments; and the incorporation of adaptive pathways to provide flexibility and resilience in a changing and uncertain environment (Wise et al. 2014).

A common element in each of the above domains is the need to address significant trade-offs and synergies for whole-of-system solutions. Some examples identified through the co-design process are shown in Box 2 (typical trade-off issues at various scales) and Box 3 (examples of strategic directions that can provide multiple benefits). Traditional business cases and financing options can be enhanced by capturing the values of such synergies and co-benefits, as well as the impact of trade-offs.

**Box 2:** Key examples of difficult urban trade-offs and choices at various scales, identified in the co-design process with stakeholders

**Regional scale**	
Urban growth vs. maintaining peri-urban/rural land uses and livelihoods	
Resolving food—energy—water nexus issues	
**Metropolitan/local council scale**	
Activity growth centres: larger number of smaller centres (more distributed) vs. smaller number of larger centres (more centralised)	
Increased density vs. pressure on local space, environment, micro-climates	
Public transport benefits vs. current/growing automobile-based road investments	
**Local council/precinct scale**	
Land use zoning and regeneration: conflicting values and vested interest pressures	
Centralised infrastructure interests vs. decentralised innovation, benefits	
Asset hazard management strategies: protect vs. accommodate vs. retreat	
**Household scale**	
Greater affluence and expectations vs. drive for smaller living/working spaces, reduced consumption,	
Climate resilient building materials vs. sustainable building materials vs. cost

**Box 3:** Key examples of synergistic opportunities identified in the co-design process with stakeholders

**Higher density, distributed activity growth centres connected through mass/electrified/integrated/active transport**—agglomeration benefits; less travel time; reduced resource use/pollution; community resilience and health benefits; economic benefits	
**Regeneration of settlements/precincts with distributed energy, water, food infrastructure and enhanced green, blue, social infrastructure**—more housing/work choice; local community ownership/cohesion; greater accessibility; lower footprint; more resilience; stronger ecosystem services; community amenity; health benefits; economic value generation	
**Reduced sprawl, preservation and improvement of hinterland/peri-urban natural and agricultural assets**—improved natural resources services, access and quality; amenity and tourism; broader economic value and livelihoods; health benefits	
**More sustainable industry, business, household resource use, consumption and waste management**—resource efficiency; less waste (food, water, energy, materials, pollution, GHG); improved diet/nutrition; multiple health benefits; economic savings	
**Direct climate adaptation measures (e.g. risk mitigation; impact cascade, contingency emergency and health services planning)**—community resilience; health benefits; reduced economic losses	
**Green growth and green business development**—innovation and opportunity; economic benefits	

This emphasises the importance of framing the strategic issues and opportunities broadly enough from the outset, and over a long enough time scale. Too narrow a framing fails to identify trade-offs and synergies. In contrast, framing that adequately encompasses the more significant synergies can simultaneously help resolve the more difficult trade-offs, facilitate whole-of-system solutions, and open the path to significant transformational change.

A final clear message from the co-design process was that practitioners seek improvement in the synthesis, translation and application of *existing* as well as *new* knowledge. This includes sector-specific knowledge, research and practice, even though the most significant knowledge gaps had been identified at the integrated systems level. Hence building on the existing research and knowledge base and capabilities is a key part of any solution, with improved platforms and approaches for mapping and translating knowledge into practice.

Overall the above findings evidence that there is no single solution, but rather the need for a strategic multi-layered approach which has the potential to facilitate systemic transformation, guiding and facilitating change through both top-down and bottom-up influences, and at various spatial, governance and temporal scales—a systemic response to systemic challenges. In addition to longer term and broader scale policies and investments, incremental and experimental approaches are seen as essential components of an overall transformational approach, not as an alternative. Priorities may vary from place to place and time to time, but future directions need to draw on the full range of complementary levers.

## Discussion and conclusions

The article has described the initial outcomes of a co-design process for sustainable urban development, drawing on a combination of Australian and international experience and research. A knowledge framework (Fig. 2) has been developed, and used in a preliminary way to explore strategic urban issues and the implications for integrated and transformational decision making in Australian cities. Not all the individual insights are new, but their combination, developed through a multi-scale co-design approach, is novel.

Consistent with the reflexive consideration of co-design outcomes indicated in Fig. 1, further insights have been identified that should help shape future directions. These cover implications for urban policy/decision making, supportive and collaborative research, the co-design process itself, and a forward agenda for collaborative knowledge development and use.

### Reflections on urban policy and decision making

#### Developing systemic, multi-faceted and multi-layered responses

We found from the Australian experience that a wide range of systemic barriers are leading to significant gaps between publicly stated goals and actual decision making and practice. Practice has tended to be siloed, with fragmented agendas and limited management of interdependencies, trade-offs and synergies. It emerged from the co-design process that policy responses to this situation will need to be systemic, multi-faceted and multi-layered, with an active seeking out of useful synergies. Such an approach is consistent with the exercise of multiple leverage points to guide the evolution of complex social–ecological systems (Abson et al. 2017), and the principles of the Multi-Level Perspective (MLP) from socio-technical transition theory (Geels 2002).

Thus the need identified for shared vision and goal development, and for more coherent and systemic policy responses at national and sub-national levels (to turn current barriers into enablers), represent systems leverage respectively at the ‘intent’ and the ‘design of social structures and institutions’ levels (Abson et al. 2017); and at the same time a redirection at the ‘landscape’ level in the MLP. In parallel with this, identified initiatives at both broader metropolitan and local scales, represent ‘niches’ that can challenge and change incumbent ‘regimes’ under the MLP, and at the same time exercise middle- and lower-level leverage on the urban systems (e.g. the ‘management of system feedbacks and parameters’).

Thus policy and decision makers may well benefit from explicit application of these frameworks. Indeed the translation of socio-technical transition theory and MLP into the urban context is starting to emerge (Berkhout et al. 2010; Hodson and Marvin 2010; Næss and Vogel 2012).

A more systemic response to the identified barriers would also benefit from ‘policy mix’ thinking, with ‘policy process coherence’ and ‘policy instrument consistency’ across multiple goals, sectors, scales and roles (Rogge and Reichardt 2016), rather than ‘individual issue/policy’ responses.

#### Broader framing of individual urban issues: At all scales

A more holistic approach also highlights the importance to policy making of broader framing of individual urban issues, to facilitate development of multi-objective solutions, and better leverage synergies to facilitate transformation. Examples of such synergies were identified through the co-design process (Box 3). These can better demonstrate the true costs and benefits to society, and facilitate financing.

To a significant extent, these framing opportunities are independent of the size and shape of a city. What is considered a city is rapidly changing in a globally and locally networked world, where the city needs to be understood as flows, interactions and processes and not just locations (Neuman and Hull 2009; Castells 2010; Batty 2013); and stakeholders come from the same sectors of society and the same multiple levels of government, regardless of city size (Healey 2006; Neuman 2007).

#### Addressing the urban planning and design dilemma

The findings highlight the dilemma for formal urban planning and design functions. On the one hand there is a clear opportunity to help shape the future, whilst on the other a growing recognition that urban futures are emergent, driven by multiple drivers that are not amenable to planned solutions in the ‘self-organising city’ (Portugali et al. 2012). While it may therefore be true that policy guidance for smaller scale local initiatives should be flexible to allow bottom-up innovation (Moroni 2015), broader scale collective decision making on critical infrastructure and public realm investment is more complex, especially when multiple objectives and interdependencies are introduced. The need for better decision support in such areas is clearly reflected in the priority decision making domains that emerged from the co-design process, and is also reflected in recent international reports (UNEP 2013).

The opportunity to better combine spatial/locational and process views of cities also lies behind calls for greater collaboration between planning and design functions and systems-oriented disciplines such as urban ecology (Childers et al. 2015).

#### Innovation in institutions, governance and engagement

Two prerequisites for such future planning are clear—continuing innovation in institutional and governance approaches, and meaningful community and stakeholder engagement. Complex urban systems are characterised by the diversity of actors, and the need for coherent and adaptive multi-level governance (Neuman 2007; Loorbach 2010; Ostrom and Cox 2010). Sustainable urban development will require significant redesign of many social, political, financial and other institutional structures over time (Young 2010), increasingly generated on a foundation of democracy, decentralisation and strong social movements and engagement (Satterthwaite 2013). A successful transdisciplinary approach also requires engagement from the outset with the full diversity of community and other stakeholder aspirations and values (Hartz-Karp and Newman 2006), including pursuit of social justice, equity and inclusion goals. Co-production of solutions-based knowledge will also require significant cultural and procedural changes at individual organisational and actor levels to recognise the importance and legitimacy of multiple societal goals, values and sources of knowledge. For example, even where extensive co-production processes have been carried out, there can be major obstacles to reintegrating the knowledge and implications back into the key organisations because of different institutional cultures, practices and mindsets (Polk 2015).

### Reflections on supportive and collaborative research

#### Taking an integrating ‘whole-of-system’ perspective

The co-design process confirmed that, in order to support policy and decision makers, collaborative research will increasingly require a whole-of-system perspective, with contributions from multiple disciplines, frameworks, methodologies and an increasing range of models and data. Only if the overall urban system and its subsystems are better understood can decision makers identify priority leverage points for transformational change, and increase the likelihood of achieving intended outcomes.

Approaches to developing a more holistic and integrated ‘science of cities’ are starting to emerge with recognition that such a science needs to integrate approaches across the local to global continuum (Pickett and Zhou 2015), and reflect certain intrinsic features of the contemporary city e.g. complexity, connectedness, diffuseness and diversity (McHale et al. 2015). More specifically, potential to combine insights from natural integrating fields is being recognised, such as urban ecology (Pickett et al. 2011; McPhearson et al. 2016b) and complex urban systems studies (Batty 2013; Bettencourt 2013a, b). Clearly this is not a trivial ambition. The knowledge framework and approach developed in our study aim to contribute to such an integrating agenda.

#### Drawing on multiple disciplines, frameworks and methodologies

This also requires the engagement of many disciplines (e.g. geographers; planners/designers/architects; engineers; ecologists; economists; social and policy scientists) brought together in inter- and transdisciplinary discourse. As mentioned, insights can also be drawn from multiple useful frameworks and methodologies (e.g. social–ecological systems (SES), resilience thinking, socio-spatial and socio-technical transition (STT) theories, and sustainability and complex systems sciences). Recent studies have helped identify common ground and synergies between many of these approaches, while recognising that each also provides distinctive insights and perspectives (Smith and Stirling 2010 on SES and STT; Anderies et al. 2013, Redman 2014, Wu 2014 and Shahadu 2016 on sustainability science, SES and resilience thinking). Another development to be encouraged is the increased translation of these into the urban context (Crawford et al. 2005 on complex systems science, spatial patterns and land use; Wilkinson 2012 on SES, resilience and urban planning; and Weinstein and Turner 2012 on sustainability science in the urban context).

#### Developing metropolitan-scale, cross-sector and behavioural models and data

Our co-design process also identified a need for greater focus on metropolitan-scale and cross-sector data and models. Urban decision makers may be familiar with the use of traditional engineering and economic modelling, but less so with complex systems dynamic models (Batty 2008, 2013; Rickwood 2011; Baynes and Wiedmann 2012), including agent-based modelling that can enhance understanding of complex behavioural drivers. There is also potential to draw on the vision of smart cities and urban analytics, with the use of new data and information sources (e.g. ‘big data’ from city infrastructure and service systems, sensors, social media), that can in turn spark new theories (Batty et al. 2012).

It is still an open question whether the full scope of urban systems and processes can be adequately described using complex systems science models, building on more limited sectoral modelling, such as that for urban development (Baynes 2009), urban water management (Moglia et al. 2010) and urban planning (Rickwood 2011). Nonetheless, at the very least such modelling can play an important role in facilitating collaboration and shared understanding with stakeholders (Guhathakurta 2002). An analysis of 17 current urban modelling systems that were designed to provide practical decision support, confirmed that none provide the full range of desired integrated capabilities (TEST 2013), leading to ongoing development of at least one attempt to fill this gap and provide an enhanced basis for collaborative urban co-design at various scales (TEST 2016).

#### Assisting in the translation of learning from others

Finally the co-design process confirmed the strong interest in learning from the practical experience of others. Researchers can contribute here by supporting innovative approaches with the potential for upscaling (Bai et al. 2010), and by facilitating comparative analysis of case studies across different cities and projects (Berkhout et al. 2010; McCormick et al. 2013). However, there is a need for a more extensive and systematic approach to clarify how the effectiveness of solutions might be linked to city typologies, profiles and local context. Such learning is especially critical to support guided and evolutionary transitions in a polycentric governance and substantially self-organising urban environment.

### Reflections on the co-production process and taking the agenda forward

It is clear that a systems approach with transformational aspirations, places even greater demand on the co-production of knowledge. Our study has effectively been a first pass, at local to national levels, of the first and second (co-design) phases of the co-production process in Fig. 1. This sets a context for the next phase of ‘Developing knowledge and solutions’.

Some reflections from the participants on the co-design process to date, with implications for future directions, include:

- Starting the engagement process primarily with government stakeholders at various levels and across jurisdictions is useful, as they are likely to take the broadest perspective and are closest to being ‘owners of the system’ on behalf of the communities they represent. They also have the potential to set a more coherent ‘multi-level’ governance framework that can facilitate the actions of other non-government decision makers.
- However, no one owns the whole system so it is necessary at an early stage to also engage with private and community sector stakeholders, even at the broad national and international agenda-setting stage. These stakeholders will bring their own values and priorities, and some will be potential collaborative partners and resource providers going forward.
- Running a good practice co-design/co-production process is no guarantee by itself that findings will be taken up in practice. It is especially a challenge to establish stakeholder ownership of complex multi-level issues, and the necessary cross-organisational leadership to challenge traditional institutional thinking. In the Australian context, and to complement and support ground-up initiatives, this will require more coherent national and sub-national government policy direction, sustained beyond short-term election cycles, and including facilitation of the multi-level processes.
- Pursuing a collaborative agenda of this nature is a long-term process, requiring sustained program management, team building, and development of relationships built on trust. As it is likely in practice to be implemented by multiple, independently funded projects, the challenge is to coordinate and integrate these efforts to progress the overall agenda. This also requires continuing commitment and shared leadership.

Whilst this has been a promising start, the aim is to continue to develop a collaborative research agenda in Australia, preferably through a sustainable urban development initiative within the Future Earth Australia program. The co-design process and findings have directly informed the potential components of such an initiative and these are summarised in Table 2, with some early suggested links to international efforts.

**Table 2:** Taking the sustainable urbanisation agenda forward (including stakeholder views from the co-design process)

Collaborative activity	Comments/examples
Collaborate with a growing network of researchers and stakeholders on the overall sustainable urbanisation approach and priority issues, building on the outcomes from co-design processes to date (including the process and knowledge frameworks at Figs. 1 and 2)	Internationally: link to international networks including Future Earth Urban Knowledge Action Network (global and regional) Australia: Build on existing collaborative programs (e.g. Cooperative Research Centres for Low Carbon Living and for Water Sensitive Cities; the ‘Visions and Pathways 2040’ project)
Map and consolidate (or link) knowledge into more integrated and accessible platforms, initially drawing on existing research and knowledge bases, drilling down from a shared overarching knowledge framework (e.g. Fig. 2)	The need to improve synthesis, translation and application of *existing* as well as *new* knowledge, was identified as crucial. This included sector-oriented knowledge, though the most significant gaps identified were at the integrated systems level
Move from co-design to co-production of new integrated knowledge in identified priority areas, through specific collaborative research projects	Priorities could for example be identified from the high leverage strategic urban issues identified (e.g. “Strategic focal areas for integrated and transformational change” section/Fig. 3 in the Australian context), with framing that includes critical trade-offs and synergies. This would advance systems-based and transformational collaborative research in specific cities and contexts
Initiating meta-studies and comparative case studies across multiple cities, to yield insights on potential solutions, and on the extent to which (or context in which) they may be transferable	Most useful when international. Through the co-design process, examples from several Australian jurisdictions were identified as potential case studies, often drawing on urban initiatives already completed or under way
Continuing to develop an overarching knowledge framework (or equivalent) through further collaborative activity	The knowledge framework at Fig. 2 evolved iteratively throughout the co-design process, and also built on other existing frameworks. It should continue to evolve in practical use, as a vehicle to enhance shared understanding and practical application

To further inform such a move from co-design to knowledge co-production, at local and broader levels, it is important to reflect on recent findings from the literature. Thus Grove et al. (2015) confirm that the complexity of co-production for sustainable urban development is different in degree from that encountered in earlier participatory research, with more, and more varied disciplines, actors and connections, operating across multiple spatial and time scales, and targeting multiple sustainability goals (Nevens et al. 2013; Bai et al. 2016). Wachsmuth et al. (2016) also indicate that urban sustainability initiatives need to be more broadly framed both spatially and socially, addressing equity and other objectives across multiple scales. These characteristics mean that knowledge systems are not just about the various sources of knowledge but also diverse networks of actors connected by often conflicting social, political, power and cultural relationships and dynamics (Munoz-Erickson 2014; Grove et al. 2015).

Drilling down, Polk (2015) provides a useful set of five knowledge co-production ‘focal areas’—*inclusion, collaboration, integration, usability and reflexivity*. We use these in what follows to synthesise challenges identified in the co-production literature. These can manifest as either barriers to or (if well handled) enablers of change.

*Inclusion* involves identifying key actor participation (for which social network analysis can assist) and an approach that encourages open discussion, common language, a free and safe environment, and continuing engagement throughout the process, even though time availability of participants is frequently a challenge (Polk 2015; Frantzeskaki and Kabisch 2016). *Collaboration*, including forming partnerships, is an additional step that requires building even greater levels of mutual trust, learning and capacity building; clear roles and rules of engagement; and, while recognising the multiple synergies and benefits of cooperation, building in approaches to manage conflict and renegotiate where new issues arise (Lang et al. 2012; Mauser et al. 2013; Polk 2015; Gorissen et al. 2016; Huchzermeyer and Misselwitz 2016). In this sense partnerships and well-designed collaborative ‘spaces’ can be seen as helping create new governance arrangements that facilitate collaboration across existing institutions and jurisdictions, while also ‘setting the scene’ for emergent solutions and innovation, and connecting longer-term visions with quick wins (Nevens et al. 2013; Frantzeskaki and Kabisch 2016).

*Knowledge integration* requires shared understanding of multiple knowledge sources. This can be challenging not only because of the need to translate between different disciplinary, professional and community ‘languages’, but also because participants are likely to bring to the table multiple and potentially conflicting framings of the issues being addressed (Lang et al. 2012; Mauser et al. 2013; Polk 2015). This, along with other factors, then conditions what are seen as legitimate sources of knowledge. The literature includes several examples where collaboration and knowledge integration issues have been addressed through shared knowledge frameworks; participative envisioning of desirable futures followed by backcasting to develop strategies and pathways that move towards such futures; and setting up collaborative experiments with the potential to take modest but immediate steps forward (e.g. Nevens et al. 2013; Frantzeskaki and Kabisch 2016). There are also warnings that the recent focus on ‘smartification’ of cities and other technical solutions will only be helpful if combined with understanding of, and engagement with, the social context within which they are proposed (Gorissen et al. 2016; Huchzermeyer and Misselwitz 2016).

Finally, *usability* and *reflexivity* are closely connected, particularly when assessing the uptake of co-produced findings beyond the participants directly involved. A minimum requirement is to ensure salience to stakeholders of the findings, which requires a solutions orientation and tailoring of communication to diverse audiences (Lang et al. 2012; Grove et al. 2015). However, even where best efforts are made in these respects, current institutional mindsets, cultures, roles and practices will often be a major barrier to the take-up of the direct findings, let alone reflection on broader change implications and opportunities for upscaling and replication (Mauser et al. 2013; Nevens et al. 2013; Polk 2015; Frantzeskaki and Kabisch 2016). While local governments can play a key role as integrators at the local level, Wachsmuth et al. (2016) conclude that national and state/provincial governments need to apply more coherent and supportive sustainability policies across local jurisdictions.

These insights are being built into our own proposed next steps, and confirm the challenges in progressing the sustainable development agenda in Australia and internationally. In Australia, there has recently been a renewed focus at the national level on a cities agenda, to address such issues as long-term and integrated planning for infrastructure; more diverse and affordable housing closer to sources of employment; encouragement of urban renewal; and alternative strategic and financing options such as value capture (Australian Government 2016). The translation of this renewed intent into supportive policy settings and investments should be an opportunity to progress sustainable urban knowledge development and use.

While we have drawn especially on the experience of Australian cities, the intent of our study has equally been to contribute to international initiatives, including the Future Earth Urbanisation Knowledge Action Network, both globally and in the Asia-Pacific region (Future Earth 2015, 2016). The Future Earth initiative’s urbanisation theme, along with other key urban networks (e.g. ICLEI, C40), provide potential international platforms to co-produce and translate relevant knowledge.

This agenda, contributing to a more integrated science of cities and sustainable urban development decision making, needs to be progressed with a sense of urgency. With much of the growth in urbanisation still to come, there is a window of opportunity to address the complex and multi-level issues from national, regional and local decision makers’ perspectives. But time is not on our side.
